# *Streptococcus iniae* SF1: Complete Genome Sequence, Proteomic Profile, and Immunoprotective Antigens

**DOI:** 10.1371/journal.pone.0091324

**Published:** 2014-03-12

**Authors:** Bao-cun Zhang, Jian Zhang, Li Sun

**Affiliations:** 1 Key Laboratory of Experimental Marine Biology, Institute of Oceanology, Chinese Academy of Sciences, Qingdao, China; 2 Graduate University of the Chinese Academy of Sciences, Beijing, China; 3 Collaborative Innovation Center of Deep Sea Biology, Zhejiang University, Hangzhou, China; University of Kansas Medical Center, United States of America

## Abstract

*Streptococcus iniae* is a Gram-positive bacterium that is reckoned one of the most severe aquaculture pathogens. It has a broad host range among farmed marine and freshwater fish and can also cause zoonotic infection in humans. Here we report for the first time the complete genome sequence as well as the host factor-induced proteomic profile of a pathogenic *S. iniae* strain, SF1, a serotype I isolate from diseased fish. SF1 possesses a single chromosome of 2,149,844 base pairs, which contains 2,125 predicted protein coding sequences (CDS), 12 rRNA genes, and 45 tRNA genes. Among the protein-encoding CDS are genes involved in resource acquisition and utilization, signal sensing and transduction, carbohydrate metabolism, and defense against host immune response. Potential virulence genes include those encoding adhesins, autolysins, toxins, exoenzymes, and proteases. In addition, two putative prophages and a CRISPR-Cas system were found in the genome, the latter containing a CRISPR locus and four *cas* genes. Proteomic analysis detected 21 secreted proteins whose expressions were induced by host serum. Five of the serum-responsive proteins were subjected to immunoprotective analysis, which revealed that two of the proteins were highly protective against lethal *S. iniae* challenge when used as purified recombinant subunit vaccines. Taken together, these results provide an important molecular basis for future study of *S. iniae* in various aspects, in particular those related to pathogenesis and disease control.

## Introduction

*Streptococcus iniae* is a Gram-positive bacterium and an important pathogen in aquaculture. It can infect at least 27 different species of economically important marine and freshwater fish including barramundi, Japanese flounder, rainbow trout, turbot, tilapia, hybrid striped bass, channel catfish, and European sea bass [1], [2]. The first confirmed streptococcal infection occurred in Japan; since then disease outbreaks and heavy economic losses due to *S. iniae* have been reported in various locations around the world, notably the United States, Israel, Canada, Japan, China, and Singapore [1]. Fish infected with *S. iniae* exhibit varied clinical signs and often develop meningoencephalitis and generalized septicaemia, which are highly lethal diseases that can cause 30–50% stock mortality [3], [4]. As a result, *S. iniae* is reckoned one of the most severe pathogens to aquaculture, in particular where intensive farming is conducted. In addition to be an infectious agent to aquaculture animals, *S. iniae* is also an opportunistic zoonotic pathogen and can cause serious infections in humans, often through transmission of *S. iniae*-contaminated fish [1], [4]. Recent studies have identified a number of virulence-associated factors that are involved in *S. iniae* infection, which include surface proteins, capsular polysaccharides, transcription regulators, and secreted protein products [5]–[14]. Nevertheless, the fundamental pathogenic mechanism of *S. iniae* remains to be investigated.

Candidate *S. iniae* vaccines in various forms, i.e. bacterins, subunit vaccines, attenuated vaccines, and DNA vaccines, have been reported, and *S. iniae* bacterins have been licensed in some countries. However, practical application of bacterins in countries such as Israel and Australia has proved to be problematic, since *S. iniae* is highly variable and, under vaccination pressure, develops variations in capsular and polysaccharide structures that enable the variant bacteria to evade vaccine-induced host immunity [15]–[20].

Although the whole-genome sequences of a number of Gram-negative fish bacterial pathogens have been reported, no fish pathogens of Gram-positive nature have been sequenced at the complete genome level. Recently, three draft genome sequences of *S. iniae* have been released (GenBank accession nos.: BANM00000000, AMOO00000000, and AOCT00000000). Of these sequences, only that of *S. iniae* 9117 (GenBank accession no. AMOO00000000), a human clinical isolate, was annotated. One intrinsic problem of shotgun sequence is the existence of large stocks of gaps. In this study, in order to accurately unravel the genetic information of *S. iniae*, we determined the complete genome sequence of *S. iniae* SF1, a pathogenic fish isolate of serotype I [21]. In addition, we also conducted proteomic analysis to identify extracellular proteins induced by host serum and examined the immunoprotective potentials of these proteins as recombinant subunit vaccines.

## Materials and Methods

### Ethics statement

Experiments involving live animals were conducted in accordance with the “Regulations for the Administration of Affairs Concerning Experimental Animals” promulgated by the State Science and Technology Commission of Shandong Province. The study was approved by the ethics committee of Institute of Oceanology, Chinese Academy of Sciences.

### Bacterial strains and growth conditions

*S. iniae* SF1 (serotype I) is a pathogenic strain that had caused an epidemic in farmed flounder [21]. It was cultured in TSAYE medium [22] at 28°C. *Escherichia coli* BL21(DE3) (used in this study as a host strain for expression and purification of recombinant proteins) was purchased from Tiangen (Beijing, China) and cultured in Luria-Bertani broth (LB) medium at 37°C.

### DNA extraction

Genomic DNA was prepared with the TIANamp Bacteria DNA Kit (Tiangen, Beijing, China). DNA was dissolved in TE buffer (10 mM Tris-HCl, 1 mM EDTA, pH 8.0). The concentration and purity of the DNA were determined with NanoDrop 2000 (Thermo Fisher Scientific, USA).

### DNA sequencing

Two different gDNA libraries were constructed according to the manufacturer's instructions of Roche GS FLX system and Illumina Miseq system. gDNA sequencing was performed on Roche 454 GS FLX Titanium chemistry (Roche Diagnostics) and Illumina Miseq (251 PE, Illumina) by Shanghai Personalbio Biotechnology (Shanghai, China). Long-insert (1 kb) libraries were sequenced using Roche FLX454 by the single end mode, and short-insert (300 bp–500 bp) libraries were sequenced with Illumina Miseq in the manner of paired end mode (2×251). The combined reads achieved about 100× coverage of the genome.

### Assembly

The reads from clean data were de novo assembled using Newbler (version 2.6) developed by 454 Life Sciences (Branford, Connecticut, USA), which yielded a number of contigs with different lengths. SSPACE (Version 2.0) [23] was used to combine contigs into scaffolds. Gaps were filled in by sequencing the PCR products using ABI 3730 capillary sequencers. Phred, Phrap, and Consed software packages (http://www.genome.washington.edu) were used for the final assembly and edition, and low quality regions of the genome were re-sequenced. To verify the assembly, the genomic DNA was digested with restriction enzymes, and the products were subjected to pulsed-field gel electrophoresis analysis.

### Sequence analysis

Putative coding sequences (CDSs) were identified by Glimmer 3.0 [24], and the length of open reading frame (ORF) was set to be more than 100 bp. Sequences from intergenic regions were compared to GenBank's non-redundant (nr) protein database [25] to identify genes missed by Glimmer. For non-coding RNAs, transfer RNA (tRNA) genes were predicted with tRNAScan-SE (version 1.3.1) [26], and ribosomal RNA (rRNA) genes were identified by RNAmmer 1.2 [27]. Functional annotation of CDSs was performed by searching against nr protein database using BLASTP [28], BLAST2GO (http://www.blast2go.com/), COG (http://www.ncbi.nlm.nih.gov/COG/) [29], and KEGG (Kyoto encyclopedia of genes and genomes; http://www.genome.jp/kegg/) [30]. The criteria used to assign function to a CDS were (i) a minimum cutoff of 40% identity and 60% coverage of the protein length, and (ii) at least two best hits in the COG, KEGG, or nr protein database. Prediction of Clustered Regularly Interspaced Short Palindromic Repeats (CRISPRs) was performed using CRISPR recognition tool (http://crispr.u-psud.fr/crispr/). Prophage analysis was conducted with PHAST (PHAge Search Tool) (http://phast.wishartlab.com/) [31]. Putative virulence factors were identified according to VFDB (http://www.mgc.ac.cn/VFs/main.htm). The complete genome sequence of *S. iniae* SF1 has been deposited in GenBank under the accession number CP005941.

### Preparation of extracellular proteins

*S. iniae* SF1 was cultured in TSAYE medium at 28°C to an OD_600_ of 0.5 and centrifuged at 5000×*g* for 5 min. The cells were washed three times with PBS and resuspended in an equal volume of PBS. Two samples, named A and B, were then prepared. Sample A consisted of 27 ml of the SF1 suspension and 3 ml turbot serum, and Sample B consisted of 27 ml of SF1 suspension and 3 ml PBS (control). To reduce the effect of serum proteins, a third sample (Sample C) was prepared, which consisted of 27 ml PBS and 3 ml turbot serum. The three samples were placed separately into dialysis bags (molecular weight cut off 3.5 kDa), and the bags were immersed in LB medium and incubated at 28°C for 12 h with gentle shaking. After incubation, the cultures were taken out of the bags and centrifuged at 10000×*g* for 30 min at 4°C. After centrifugation, the supernatants were collected. The supernatant from Sample A was hereafter referred to as serum-treated SF1 sample; the supernatants of Samples B and C were mixed, and the mixture was hereafter referred to as untreated SF1 sample. All supernatants were filtered with 0.22 μm filters (Millipore, Billerica, MA, USA) and concentrated with PEG 20000 (Solarbio, Beijing, China) to 3 ml. The proteins were treated with 10% TCA-acetone at −20°C for overnight and then centrifuged at 15000×*g* for 1 h at 4°C. The precipitated proteins were washed with 90% cold acetone and dissolved in UTCD solution (7 M urea, 2 M thiourea, 2% CHAPS, and 40 mM DTT). The proteins were further purified with 2D-Clean-Up Kit (GE Healthcare, Piscataway, NJ, USA), and the concentrations of the proteins were determined using BCA Protein Assay Kit (Sangon Biotech, Shanghai, China).

### Two-dimensional gel electrophoresis (2-DE)

The isoelectric focusing (IEF) was carried out using Ettan IPGphor 3 system (GE Healthcare, Piscataway, NJ, USA). The protein loading volume was adjusted to 0.6 mg/ml with IEF sample loading solution (7 M urea, 2 M thiourea, 2% CHAPS, 40 mM DTT, 0.5% IPG buffer, 0.002% bromophenol blue). Two-DE was performed as reported previously [32]. The gel images were acquired using ImageScanner III (GE healthcare, Piscataway, NJ, USA) and analyzed with ImageMaster 2D Platinum 6.0 (GE healthcare, Piscataway, NJ, USA). Triplicate runs were conducted for each sample to ensure reproducibility. For comparative analysis, the percentage intensity volume (%vol) of each spot was used for comparison of matched spots between serum-treated and untreated samples. To reduce potential errors, a ratio of ≥2 (or ≤0.5) and analysis of variance (ANOVA) <0.05 were taken as a threshold for differential expression. The differentially expressed protein spots were picked from the gels and washed first with water and then with 25 mM ammonium bicarbonate in 50% acetonitrile for 60 min. The gel spots were processed by enzymatic digestion and analyzed by matrix-assisted laser desorption/ionization time of flight (MALDI-TOF) mass spectrometry as reported previously [32].

### Purification of recombinant proteins

In order to obtain purified recombinant enolase (Eno), neuraminidase (Neu), Fe^3+^-siderophore transport protein (Stp), hemolysin (Hem), and hypothetical secreted protein (Hyp1), the plasmids pEno, pNeu, pStp, pHem, and pHyp1 were constructed, which express His-tagged Eno, Neu, Stp, Hem, and Hyp1 respectively. For this purpose, the coding sequences of Eno, Neu, Stp, Hem, and Hyp1 were amplified by PCR with the primer pairs EnoF1/EnoR1, NeuF1/NeuR1, StpF1/StpR1, HemF1/HemR1, and Hyp1F1/Hyp1R1 respectively (Table 1). The PCR products were ligated to the TA cloning vector (Tiangen, Beijing, China), and the recombinant plasmids were digested with EcoRV or SmaI to retrieve the fragments containing the PCR inserts, which were inserted into pET259 [33]at the SwaI site, resulting in pEno, pNeu, pStp, pHem, and pHyp1. *E. coli* BL21(DE3) was transformed separately with each of the plasmids, and the transformants were cultured in LB medium at 37°C to mid-log phase. Isopropyl-β-D-thiogalactopyranoside was added to the culture to a final concentration of 1 mM (for rNeu, rStp, and Hyp1), 0.4 mM (for rEno), or 0.7 mM (for rHem). The growth was continued at 30°C for an additional 4 h, and the recombinant proteins were purified using nickel-nitrilotriacetic acid columns (GE Healthcare, USA) as recommended by the manufacturer. The purified proteins were dialyzed for 24 h against PBS and treated with Triton X-114 to remove endotoxin as reported previously [34]. The proteins were concentrated with Amicon Ultra Centrifugal Filter Devices (Millipore, Billerica, MA, USA). The concentrations of the purified proteins were determined as described above. The proteins were analyzed by sodium dodecyl sulfate-polyacrylamide gel electrophoresis (SDS-PAGE) and visualized after staining with Coomassie brilliant blue R-250.

**Table 1:** Primers used in this study.

Primers	Sequences (5′ → 3′)^a^
Hyp1F1	CCCGGG ATGACTTTAAAAAAGAAATTAGTG (SmaI)
Hyp1R1	CCCGGG AATCAAACTTCTAAAGAAGTCG (SmaI)
NeuF1	GATATC ATGGAACTTTCAGATAAACA (EcoRV)
NeuR1	GATATC AAAAATTTCCAATTAAAGGT (EcoRV)
StpF1	CCCGGG ATGTTAAAAGAAGGCACC (SmaI)
StpR1	CCCGGG ATGTTAAAAGAAGGCACC (SmaI)
HemF1	GATATC ATGCCTAAAGAAAGAGTAGAT (EcoRV)
HemR1	GATATC TTCTTCTTCATGTCGATTAA (EcoRV)
EnoF1	CCCGGG ATGTCAATTATTACTGATGTTTAC (SmaI)
EnoR1	CCCGGG TTTTTTAAGGTTGTAGAATGAT (SmaI)

a Underlined nucleotides are restriction sites of the enzymes indicated in the brackets at the ends.

### Fish

Clinically healthy turbot (*Scophthalmus maximus*) (average 10.2 g) were purchased from a local fish farm and acclimatized in the laboratory for two weeks before experimental manipulation. Fish were fed daily with commercial dry pellets and maintained at ∼20°C in aerated seawater. Before experiment, fish (6%) were randomly sampled for the examination of bacterial recovery from blood, liver, kidney, and spleen, and no bacteria could be detected from any of the examined tissues of the sampled fish.

### Vaccination

Purified rEno, rNeu, rHyp1, rStp, and rHem were each resuspended in PBS to a concentration of 300 μg/ml and mixed at an equal volume with aluminum hydroxide as reported previously [35]. As a control, PBS was also mixed similarly with aluminum hydroxide. Turbot (as described above) were divided randomly into six groups (N = 50) and injected intraperitoneally (i.p.) with 100 μl of each of the proteins and the PBS control respectively. At four weeks post-vaccination (p.v.), thirty three fish were taken from the tank and challenged via i.p. injection with 100 μl *S. iniae* in PBS (5×10^8^ CFU/ml). The fish were monitored for mortality for at least 15 days. Dying fish were randomly selected for the examination of bacterial recovery from liver, kidney, and spleen. Relative percent survival (RPS) was calculated according to the following formula: RPS  =  {1 – (% mortality in vaccinated fish/% mortality in control fish)} ×100. The vaccination experiment was conducted first in a single trial without replication and then repeated in three replicates at a different time with a different batch of fish. The mean mortality and RPS values of the four vaccination trials were given in the results.

### Enzyme-linked immunosorbent assay (ELISA)

Sera were collected from vaccinated and control fish at one month p.v. and diluted 10 fold. ELISA was performed as reported previously [36].

### Statistical analysis

All statistical analyses were performed using analysis of variance (ANOVA) in SPSS 17.0 package (SPSS Inc., Chicago, IL, USA). Chi-square test with Yates' correction was used for mortality analysis, and analysis of variance (ANOVA) was used for all other analyses. In all cases, the significance level was defined as *P*<0.05.

## Results and Discussion

### General features of the SF1 genome sequence

**Figure 1:**
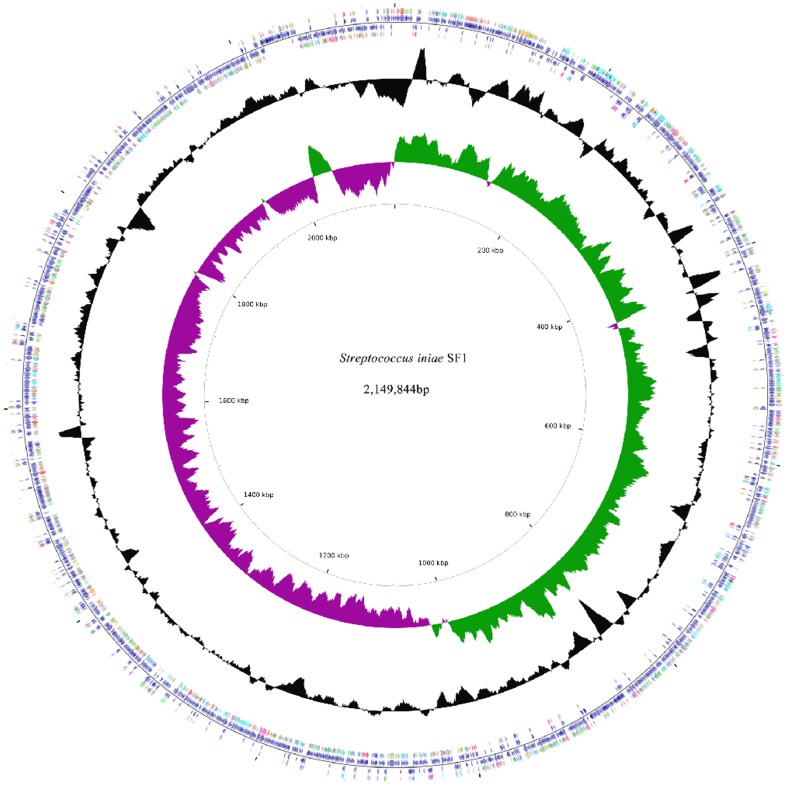
Circular maps of *Streptococcus iniae* SF1 genome. Circles range from 1 (outer circle) to 3 (inner circle) for chromosome. Circle 1, CDS (blue), tRNA (reddish brown) and rRNA (light purple); Circle 2, GC content (black); Circle 3, GC skew+ (green) and GC skew– (dark purple).

The general features of the *S. iniae* SF1 genome are summarized in Table 2. The bacterium contains a single circular chromosome of 2,149,844 base pairs with an average GC content of 36.7%. These features are similar to *S. iniae* IUSA-1, whose draft genome sequence is 2.22 Mb with an average GC content of 37.3% [37]. Low GC content appears to be a common feature of *Streptococcus* and has been observed in *S. pyogenes* (38.5%) [38], *S. pneumoniae* (39.7%) [39], and *S. agalactiae* (35.6%) [40]. The coding region accounts for 88.9% of the chromosome and is composed of 2196 coding sequences (CDS), of which 2125 are protein coding sequences. A total of 45 tRNA genes and 12 rRNA genes were found in the genome, the latter being grouped into four operons. Of the putative CDSs, 1999 (92.6%) have predicted biological functions, of which 98 (4.5%) are hypothetical proteins with similar counterparts in other genomes, and 62 (2.9%) have no substantial similarity with known proteins. Figure 1 displays the chromosome in a circular map with transcription emanating in both directions from a specific site, the origin of replication (*oriC*). It is known that GC-skew is positive in the leading strand and negative in the lagging strand [41]. In SF1, the GC-skew change was observed predominately at the origin and terminus of replication (Fig. 1), where the leading strand becomes the lagging strand and vice versa. However, an obvious positive-to-negative change occurs at the left side of the *oriC*, which may be a mark of recent genetic rearrangement such as sequence inversion or integration of foreign DNA as observed in other bacterial chromosomes [42]. In most bacteria, the majority of genes are encoded in the leading strand [43]; similarly, a strong bias in gene orientation was observed in the genome of SF1, as 76.9% of the CDS are transcribed in the same orientation as the movement of the replication fork. Leading strand-encoded genes account for 79.2% and 74.9% of the genes transcribed in the clockwise and counterclockwise directions respectively, from oriC to the replication terminus.

**Table 2:** General features of the genome of *Streptococcus iniae* SF1.

Category	Characteristics
Genome size (bp)	2,149,844
GC content (%)	36.7
Gene number	2,196
mRNA gene number	2,125
Hypothetical protein	358
tRNA	45
rRNA operon	12
Coding region (bp)	1,884,186
Coding region (%)	88.9

### CRISPR/Cas systems

**Figure 2:**
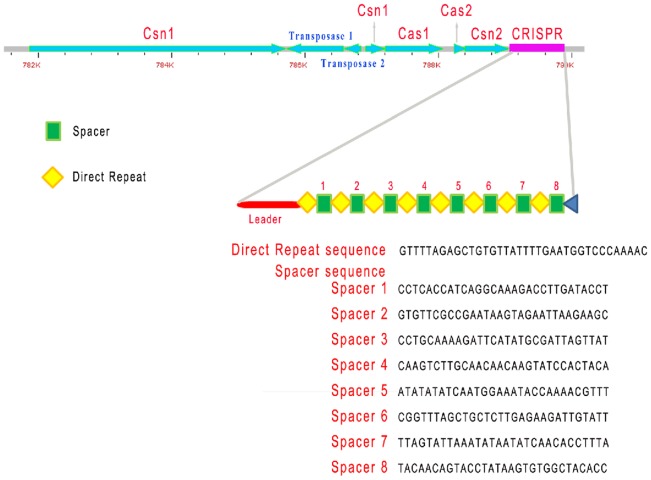
The CRISPR locus of *Streptococcus iniae* SF1. The CRISPR element is presented in pink and the CRISPR associated genes are presented in light blue. Within CRISPR, the diamonds represent complete repeat, the triangle represents incomplete repeat, and the squares indicate spacer.

Clustered regularly interspaced short palindromic repeats (CRISPR) are widespread across bacteria and archaea. They are a type of bacterial immune system that protects the cells against foreign genetic elements such as plasmids and phages. A CRISPR locus was detected in the chromosome of SF1, which is closely linked to four upstream *cas* genes (Fig. 2). The CRISPR element is composed of nine direct repeats separated by eight spacers, which are 30 bp in length and differ in sequence. Of the nine repeats, eight are identical and consist of 36 bp palindromic sequence (named complete repeat). Compared to the first eight repeats, the ninth repeat is shorter and consists of only the first 23 bp sequence of the complete repeat. The complete repeats exhibit high levels of sequence identities (89.3%–94.4%) with those identified in other *Streptococcus* sp. [44], while none of the eight spacers shows significant homology with any sequence in GenBank database. Variation in spacer regions has been observed before and used to distinguish bacterial strains/species in *Streptococcus* [45], [46]. In SF1, there is a leader region in the CRISPR locus, which is a 105 bp sequence with a high content of adenine and thymine (Fig. 2). Previous studies showed that the leader region plays an important role in acquisition of new spacers [47] and can also act as a promoter to facilitate the transcription of the CRISPR locus [48]. In the upstream of CRISPR in SF1, four *cas* genes were identified, i.e. *cas1, cas2,* two *csn1* (corresponding to *cas5*), and *csn2* (equivalent to *cas7*). These genes are known to encode proteins with functional domains involved in interaction with nucleases, polymerases, helicases, and nucleotide-binding proteins [49]–[51]. It is noteworthy that there are two transposase inserts in the *csn1* gene, resulting in two Csn1 proteins of 1281 amino acids and 99 amino acids in length respectively. Interestingly, this phenomenon is not observed in the shotgun genome sequence of *S. iniae* 9117 (GenBank accession no. AMOO00000000), in which only one *csn1* family CRISPR-associated protein with 1368 amino acid residues was detected. These observations suggest that the insertion in the *csn*1 gene of SF1 is likely an evolutionally recent event.

### Prophage

Two prophages, one questionable (848487 bp to 893083 bp) and one incomplete (2019204 bp to 2059466 bp), are harbored in the genome of SF1. The questionable phage is 44.5 kb in size, which contains 66 ORFs and has a GC content of 35.24%. It shares limited homology with other phages that have been confirmed to date; however, a large number of phage-related genes with important roles in phage life-style (replication, partition, and transfer) were identified in the questionable phage. For example, the K710_0871- encoded protein is similar to phage integrase that mediates unidirectional site-specific recombination between the phage attachment site and the bacterial attachment site [52], K710_0882 and K710_0887-89 encode putative DNA replication proteins, and K710_0875 encodes an antirepressor that is required for inactivation of the phage repressor and may regulate transfer of mobile genetic elements [53]. Meanwhile, a late gene cluster involved in DNA packaging and capsid and tail morphogenesis [54] is represented by K710_0899 to K710_0919. The incomplete phage is 45 kb in length (2019204 bp to 2059466 bp), which contains 54 CDSs and has a GC content of 37.9%. The region between 15740 bp and 27903 bp shows a significant genomic synteny with the bacteriophage PH10 of *Streptococcus oralis* [55]. Like PH10, the genome of the incomplete phage is modularly organized, including modules involved in DNA replication and DNA-packaging, head and tail morphogenesis, and cell lysis. In SF1, a DNA N-4 cytosine methyltransferase is present, which suggests the existence of a possible defense mechanism against attack from host-encoded restriction/modification systems. K710_2116 exhibits sequence similarity to the putative holin of the *Streptococcus* phage LYGO9 (GenBank accession no. AFQ95954). K710_2118 is predicted to encode a muramidase with a high level of similarity (88%) to the lysin of the prophage LambdaSa2 of *Streptococcus agalactiae* 2603 [56], [57]. These two proteins are known to be essential for host lysis, the former degrading the cell wall and the latter controlling the length of the infective cycle of lytic phages [58].

### Two-component signal transduction system

Two-component signal transduction systems (TCS) of bacteria consist of a sensor histidine kinase (HK) and a response regulator/transcription factor (RR). They play important roles in detecting and responding to diverse environmental and cellular changes/stresses [59]. Based on BlastP analysis and conserved domains of known HKs and RRs, we annotated 19 *hk* genes and 16 *rr* genes in SF1, which form 16 pairs of TCS (Table 3). In each pair, the *hk* gene and the *rr* gene reside adjacent to each other on the chromosome. Based on the homology-box and the topology feature of HK and the architecture of the C-terminal output domain of RR [60]–[62], 13 TCS pairs of SF1 are grouped into six previously described subfamilies. Two HK/RR pairs (encoded by K710_0220/K710_0221 and K710_1651/K710_1650) belong to the NarL subfamily, seven HK/RR pairs (encoded by K710_0984/K710_0983, K710_1396/K710_1397, K710_1424/K710_1425, and K710_2170/K710_2171) are of the OmpR subfamily, and two HK/RR pairs (encoded by K710_0333/K710_0334 and K710_0525/K710_0526) belong to the YesN subfamily. The five remaining HK/RR pairs fall into five different subfamilies, i.e. NtrC (K710_0267/K710_0266), LytR (K710_0405/K710_0406), CitB (K710_1119/K710_1120), YcbB (K710_1925/K710_1926), and AgrA (K710_1964/K710_1962). According to gene homology and KEGG pathway analysis, the two HK/RR pairs in the NarL subfamily appear to be orthologs of DesK/DesR and LiaS/LiaR respectively. DesK/DesR has been reported to be involved in thermosensing and signal transduction at low temperatures and can influence membrane lipid fluidity in *Bacillus subtilis* [63], [64]. Four HK/RR pairs in the OmpR subfamily were identified to be corresponding to CiaH/CiaR, PhoR/PhoB, VicK/VicR, and SaeS/SaeR respectively. Of these, CiaH/CiaR is widely distributed in other streptococcal species and known to participate in biofilm formation, stress tolerance, and subversion of host defense [65]–[67]. For the VicK/VicR pair, previous studies indicated that it plays an important role in growth, adhesion, biofilm formation, oxidative stress tolerance, development of genetic competence, and regulating intracellular pH homeostasis in *S. mutans* [68]–[70]. The two TCS pairs sorted into the YesN subfamily are homologous to the YesM-YesN of *B. subtilis* [71], whose function is not clear. The only TCS pair of SF1 that belongs to the AgrA subfamily is homologous to the AgrC/AgrA of *S. aureus*, which is known to control bacterial virulence in a cell density-dependent manner [72]. Like the *agr* operon of *S. aureus*, which contains four genes, i.e. *agrC, agrA, agrB* and *agrD*, the *agr* operon of SF1 possesses four genes, two of which are equivalents of *agrC* and *agrA*, while the other two (K710_1963 and K710_1965) have no homology to *agrB* or *agrD* but encode proteins with histidine kinase-like ATPase at the C-terminus as AgrC. It will be interesting for future studies to find out whether these two proteins can function as HKs and coordinate with AgrA as a functional TCS pair.

**Table 3:** Two-component signal transduction systems in *Streptococcus iniae* SF1.

TCS gene (hk/rr)	HK-RR	Subfamily	Putative function
K710_0220/K710_0221	DesK-DesR	NarL	Membrane lipid fluidity regulation
K710_0250/K710_0249	-	OmpR	Unknown
K710_0267/K710_0266	NtrY-NtrX	NtrC	Nitrogen regulation
K710_0333/K710_0334	YesM-YesN	YesN	Unknown
K710_0405/K710_0406	LytS-LytR	LytR	Cell autolysis
K710_0525/K710_0526	YesM-YesN	YesN	Unknown
K710_0984/K710_0983	CiaH-CiaR	OmpR	Stress resistance and pathogenesis
K710_1034/K710_1033	-	OmpR	Unknown
K710_1119/K710_1120	SivS-SivR	CitB	Regulation of capsule expression
K710_1396/K710_1397	PhoR-PhoB	OmpR	Phosphate starvation response
K710_1424/K710_1425	VicK-VicR	OmpR	Regulation of intracellular pH
K710_1605/K710_1606	-	OmpR	Unknown
K710_1651/K710_1650	LiaS-LiaR	NarL	Cell wall stress response
K710_1925/K710_1926	GlnK-GlnL	YcbB	Glutamine utilization
K710_2170/K710_2171	SaeS-SaeR	OmpR	Virulence regulation
K710_1964/K710_1962	AgrC-AgrA	AgrA	Adhesin, invasive factor

### Metabolism

**Figure 3:**
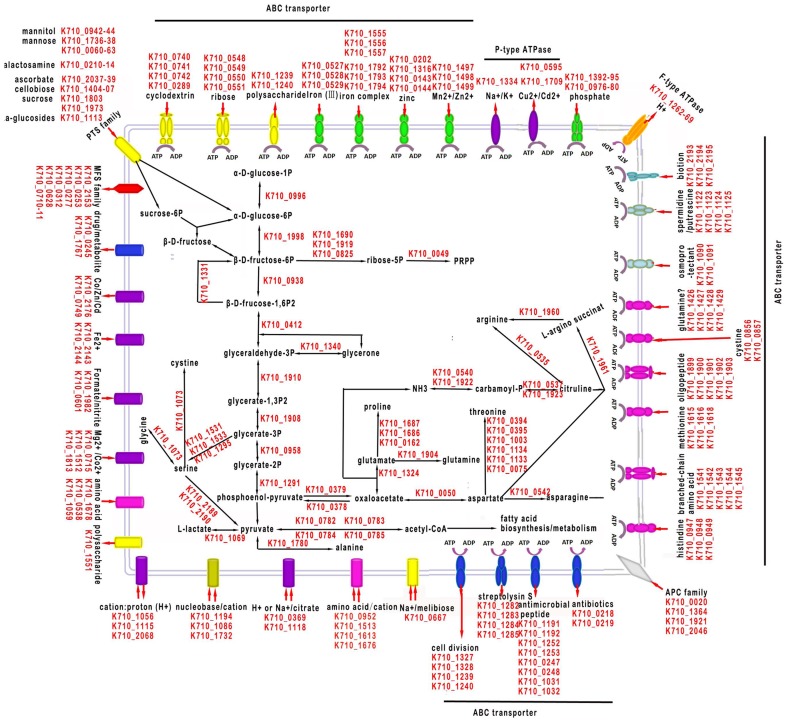
Overview of the metabolic pathways and transport systems in *Streptococcus iniae* SF1. Metabolisms and transports of sugar and organic compounds are shown. Transporters are grouped by substrate type: yellow, carbohydrates; green or purple, inorganic cations; pink, amino acids/peptides; blue, drugs or others.

An overview of the basic metabolic pathways of SF1 is presented in Figure 3. Like other *Streptococcus* species [73], SF1 appears to be able to utilize a wide variety of carbohydrate sources. Numerous genes for transport and metabolism of cyclodextrin, ribose, glucose, sucrose, β-glucoside, lactose, cellobiose, mannitol, mannose, trehalose, N-acetyl-galactosamine, and fructose were found in the genome. In bacteria, glycolytic pathway and gluconeogenesis pathway are important for carbohydrate metabolism, the former being the principal source of energy production, and the latter offering a route of transforming non-sugar materials to sugar substances. The efficiency of the glycolytic pathway is controlled by three rate-limiting enzymes, i.e., glucokinase, 6-phosphofructokinase, and pyruvate kinase, which keep a balance state of sugar supply and demand. In SF1 these three enzymes are encoded by K710_0560, K710_ 0938, and K710_0939 respectively. Pyruvate is one of the most important fermentation products and plays a key role in many metabolic pathways in a manner that depends on the specific living environments of the bacteria. Analysis the genome of SF1 showed that the pyruvate from glycolysis could be converted into lactic acid under the catalysis of D- or L-lactate dehydrogenase (encoded by K710_1069). This reaction can meet the need of *S. iniae* for ATP under anaerobic condition through production of oxidized NAD^+^. On the other hand, since SF1 lacks a pyruvate oxidase gene, it cannot turn pyruvate directly into acetate in aerobic environments. Similarly, all three key enzymes involved in gluconeogenesis are present in the genome of SF1, i.e., pyruvate carboxylase, phosphoenol-pyruvate carboxykinase, and fructose-1,6-bisphosphatase (encoded by K710_0378, K710_0379, and K710_1331 respectively). However, the gene of glucose-6-phosphatase is absent in the genome of SF1, which suggests that SF1 does not have the capacity to catalyze D-glucose-6P to D-glucose. Thus, D-glucose-6P from gluconeogenesis can only go into the pentose phosphate pathway, or be turned into D-glucose-1P with the action of phosphoglucomutase (encoded by K710_0996) and enter into starch or sucrose metabolism. In addition, enzymes in the phosphate pathway, such as glucose-6-phosphate isomerase, ribulose-phosphate 3-epimerase, and ribose-phosphate pyrophosphokinase, are also available in SF1. This pathway is the most important source of sugar molecules with different structures and facilitates conversion of different monosaccharides [74].

Like *S. pneumoniae* R6 [75], SF1 possesses no genes encoding enzymes of the citrate acid cycle, which implies that SF1 is probably not able to generate energy from the TCA cycle. However, SF1 possesses the enzymes involved in arginine deiminase pathway, i.e. arginine deiminase (encoded by K710_0535), ornithine carbamoyltransferase (encoded by K710_0537 and K710_1923), and carbamate kinase (encoded by K710_0540 and K710_1922). The arginine deiminase pathway has been discovered in a variety of bacteria; it is induced by arginine under anaerobic conditions and regulated by catabolite repression [76]. It is likely that this pathway in SF1 is a compensation for the inability of the bacterium to generate energy from the TCA cycle.

The amino acid metabolism of SF1 is shown in Figure 3. Serine biosynthesis is achieved by three enzymes, i.e. SerA, SerC, and SerB (encoded by K710_1531, K710_1533, and K710_1295 respectively). Alanine is formed by transamination through the action of alanine transaminase (encoded by K710_1780). The biosynthesis pathways from pyruvate to valine, leucine, and isoleucine are cut off because the genes of related enzymes are not present in SF1. Aspartate is synthesized via the enzymes pyruvate carboxylase (encoded by K710_0378) and aspartate aminotransferase (encoded by K710_1324). Aspartate then serves as the basis for the anabolism pathway of threonine, asparagines, and arginine. The aspartate aminotransferase encoded by K710_1324 can also catalyze conversion of 2-oxoglutarate to glutamate, which is utilized in the biosynthesis of glutamine, arginine, and proline.

### Transport

SF1 genome contains 220 genes associated with various transport systems, accounting for 10.2% of the total ORFs. Identified transporters are listed in Figure 3, of which 81.3% are ATP-dependent. Three types of solute transporting ATPases are present: P-type, F-type, and ABC-type. The P-type ATPase is a ubiquitous family of proteins involved in active pumping of charged substrates across biological membranes [77]. Of this type of ATPase, K710_1334 is predicted to encode a cation-transporting ATPase (putative calcium-transporting), and K710_595 and K710_1709 encode P-type ATPases conferring resistance to the toxic metals cadmium and copper respectively. Like *S. mutans* [78], SF1 possesses a complete F-type proton ATPase, which is encoded by the genes K710_1262 to K710_1269. F-type ATPase (F0F1 ATPase) can use an electrochemical gradient of H^+^ or Na^+^ to synthesize ATP, or hydrolyze ATP to reverse an electrochemical gradient for sodium ion/proton exchange [79]. Compared to F-type and P-type ATPases, ABC-type ATPases are most abundant in SF1. A total of 80 ABC-type transporters were identified in the genome of SF1, which accounts for 9% of the total CDSs. Twenty-nine of these transporters were unambiguously classified as importers, while the rest were presumed to be exporters. Importers are found widely in prokaryotes and usually associated with a high-affinity extra-cytoplasmic substrate binding protein (SBP), which is maintained by an N-terminal lipo-amino acid anchor at the vicinity of the cytoplasmic membrane of Gram-positive bacteria [80]. In SF1, 29 ABC-type transporters were found to contain SBP.

ABC transporters exhibit specificity to a broad range of substrates: carbohydrates, amino acids, inorganic ions, multidrug, oligopeptides, and osmoprotectants. It is interesting that ABC transporter with DNA as a substrate was not found in SF1. Of all the ABC transporters identified in SF1, twenty four are multidrug transporters. Owing to their ability to exude toxic compounds, multidrug transporters function to improve bacterial tolerance against antimicrobial substances such as antibiotics [81]. Consequently, understanding the molecular basis of multidrug permeases may assist development of efficient strategies to control bacterial infection. In SF1, twelve ABC transporters related to inorganic ion transport were detected, including one manganese transporter (encoded by K710_1497-99), which is known to be associated with virulence [82], three ferric iron transporters (encoded by K710_1555-57, K710_1792-94, and K710_527-29), and two phosphate ABC transporters (encoded by K710_0976-80 and K710_1392-95). The manganese and zinc ABC transporters are orthologous to the *znu* system, which exhibits essentially equal specificities for Zn^2+^ and Mn^2+^ [83]. There are two types of iron transporters in SF1, one, which includes two transporters (encoded by K710_1555-57 and K710_1792-94), is homologous to siderophore-dependent iron acquisition systems that bind ferrichrome as a substrate, and the other (encoded by K710_527-29) displays homology to free iron transporters that transport ferric iron from the periplasm to the cytosol. It is possible that different types of transporters may provide a flexibility for SF1 in the acquisition of iron from multiple sources. In addition, an incomplete ferrous iron transporter, Feo (Ferrous iron transport), that lacks the Feo C protein was found in SF1. Feo is known to play a role in iron uptake under the anaerobic-microaerophilic conditions of the gastrointestinal tract and thus influence the ability of bacteria to colonize the gut [84].

SF1 possesses a variety of ATP-driven amino acid transporters, as well as transporters concerned with polyamines and betaines uptake. Fifteen ABC transporters functioning in conveying amino acids and their derivatives were annotated in SF1 genome, including polar and unpolar amino acid ABC transporters, branched-chain amino acid transporters, and dipeptide/oligopeptide transporters. Besides ATP-driven ABC transporters, four APC transporters (encoded by K710_0020, K710_1364, K710_1921, and K710_2046) and six major facilitator superfamily (MFS) transporters (encoded by K710_0253, K710_0277, K710_0312, K710_0628, K710_0710-11, and K710_2153) belonging to ion motive secondary carriers [79] were also found in SF1. A four-gene operon (K710_1122-25) encodes an ABC transporter for putrescine and spermidine was identified in the genome of SF1. These polyamines are known to have pleiotropic effects on protein synthesis and cell growth in many bacteria [85]. With respect to carbohydrate transportation, only two ABC transporters were found in SF1 (Fig. 3). In contrast, nine sugar phosphotransferase systems (PTSs) were identified in SF1, which are known to function in transmembrane transport of simple sugars, low-molecular-weight derivatives of sugars, and macromolecular sugar products.

### Virulence factors

**Figure 4:**
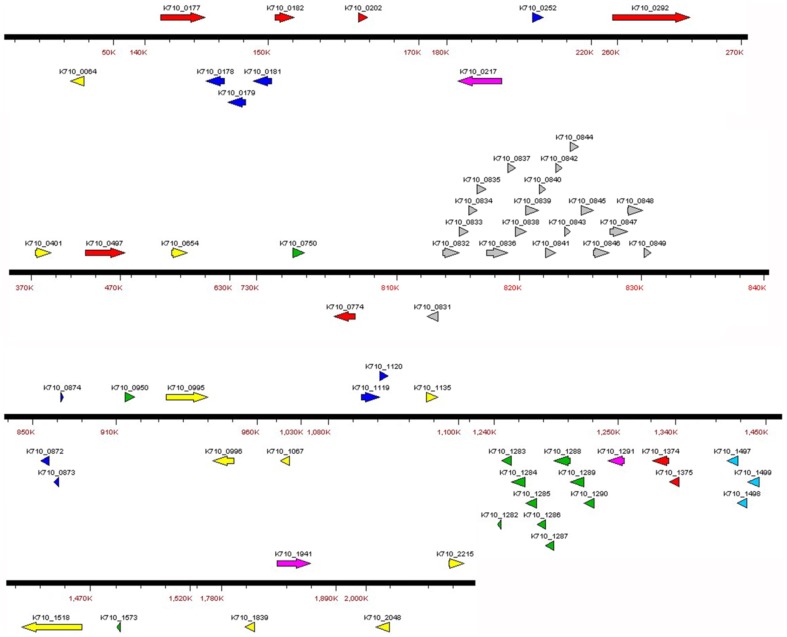
Sketch of putative virulence genes in *Streptococcus iniae* SF1. Genes were grouped into different classes marked by different colors. Yellow, protease; Red, adherence; Grey, capsule; Blue, regulation; Pink, exoenzyme; Green, toxin; Bright blue, iron uptake system.

Virulence factors enable bacteria to invade into host cells and maintain their ecological niches *in vivo*. Discovering virulence factors is essential for understanding bacterial pathogenesis and for drug and vaccine development. In this study, we identified candidate virulence genes of SF1 using BlastP and virulence factor database (VFDB), the latter dividing virulence factors into four categories: offensive, defensive, nonspecific, and regulatory [86]. Figure 4 is a schematic picture of the virulence factors identified in SF1, which include adhesins, toxins, proteases, and regulators of virulence-associated genes. All putative virulence factors are listed in Table 4.

**Table 4:** Putative virulence factors of *Streptococcus iniae* SF1.

Subclass	Protein	Gene ID
Adherence	Serine-rich glycoprotein adhesin	K710_0177
	SiM protein	K710_0182
	Lmb	K710_0202
	Alkaline amylopullulanase	K710_0292
	Agglutinin receptor	K710_0497
	FbsA	K710_0774
	Collagen binding proteins	K710_1374-75
Toxin	Exfoliative toxin	K710_0750
	CAMP factor	K710_0950
	Streptolysin S	K710_1282-90
	Alpha-hemolysin	K710_1573
Antiphagocytosis	Capsule	K710_0831-49
	Phosphoglucomutase	K710_0996
Serum resistance	Immunoglobulin-binding protein	K710_2048
Autolysin	Muramidase	K710_1175-77
	Muramidase	K710_1350
	Muramidase	K710_1760
	Amidase	K710_0064
	Amidase	K710_1996
	Transglycosylase	K710_1071
Protease	Zn protease	K710_0014
	Trigger factor	K710_0401
	Peptidase c5	K710_0995
	Sortase	K710_1067
	Clp protease	K710_1149
	Clp protease	K710_1549
	IL-8 protease	K710_1518
	C3-degrading proteinase	K710_1839
	Serine protease	K710_2215
Exoenzyme	Hyaluronatelyase	K710_0217
	Enolase	K710_1291
	Neuraminidase	K710_1941
	Streptodornase	K710_1783
Regulation	RofA/Nra-like transcriptional regulator	K710_0178
	Mga-like regulatory protein	K710_0179-81
	Rgg family transcriptional activator	K710_0252
	Toxin–antitoxin loci	K710_0872-74

#### (i) Adhesin

In Gram-positive bacteria, the majority of adhesins belong to surface proteins anchored to the cell wall via a C-terminal LPxTz motif [87]. Surface-associated adhesins and surface components recognizing adhesive matrix molecules (MSCRAMMs) can mediate colonization of Group A streptococci (GAS) in host tissues in the early stages of infection [88]. In SF1, eight putative proteins associated with adherence were identified (Fig. 4, Table 4). The protein encoded by K710_0774 is similar to the fibrinogen (Fn)-binding proteins Fbp and FbsA of *S. pyogenes* and *S. agalactiae* respectively. Fn-binding protein is known to attach bacteria to the extracellular matrix of the host, the latter acting as a bridge between the invading bacteria and host cells [87]. Like all serine-rich glycoprotein (Srr), the Srr adhesin in SF1 (encoded by K710_0177) contains two serine-rich regions and possesses nonapeptide repeats with a consensus sequence of SESMSTSES. The C-terminal repetitive region of Srr is followed by the cell wall anchor sequence LPxTG. Another adhesin with LPxTG motif is alkaline amylopullulanase (encoded by K710_0292), which has been reported to promote *S. suis* adhesion to porcine epithelium and mucus [89]. In addition, agglutinin receptor (encoded by K710_0497), collagen binding proteins (encoded by K710_1374 and K710_1375), and laminin-binding protein (encoded by K710_0202) are also recognized as potential adhesins in SF1.

#### (ii) Capsule, autolysin, and protease

The capsule locus of SF1 is composed of 19 genes encoded by K710_0831 to K710_0849 (Fig. 4). Except for the absence of an IS element between *cpsL* and *cpsM*, the genetic organization of the capsule region of SF1 is the same as that of *S. iniae* 9117 (GenBank accession no. AY904444). Several putative autolysins have been identified in SF1, which include three muramidases, two amidases, and one transglycosylase (Table 4). These proteins are implicated in many biological processes associated with the virulence of pathogenic *Streptococci*, notably cell separation, cell wall turnover and restructuring, and bacterial autolysis induced by adverse physiological conditions [90]–[92]. Protease is a type of virulence factor that degrades the proteins and peptides of the host. Some proteases of *S. iniae* have been reported to contribute to pathogenicity by breaking down host immunoproteins [93]. In SF1, various virulence-associated proteases have been identified, including C5a peptidase and IL-8 protease, both which are known to be required for *S. iniae* infection [4], C3-degrading protease, ATP-dependent Clp protease, serine protease, and Zn protease (Table 4, Fig. 4). In addition, a trigger factor (encoded by K710_0401) was found in the genome of SF1, which is known to be essential for the secretion and maturation of the cysteine protease of pathogenic *S. pyogenes* [94], [95].

#### (iii) Toxin and exoenzyme

The genome of SF1 contains the gene of CAMP factor (K710_0950), which is a toxin that damages host cell membranes and binds to the Fc fragment of immunoglobulin [96], [97]. Exfoliative toxin is another toxin identified in SF1 (encoded by K710_0750). Little study has been performed on this protein in streptococcus. In *Staphylococcus aureus*, it was reported to be an endopeptidase that can specifically cleaves desmoglein [98]. In addition, SF1 possesses both alpha-hemolysin (K710_1573) and beta-hemolysin (K710_1282-90), which is in line with the observation that disease-associated *S. iniae* isolates are largely hemolytic [8]. Four exoenzyme genes were identified in SF1, i.e., enolase, hyaluronate lyase, streptodornase, and neuraminidase (encoded by K710_1291, K710_0217, K710_1783, and K710_1941 respectively). Enolase is both a cytoplasmic protein involved in metabolic pathway and a secreted protein that binds plasminogen and contributes to infectious processes [99], [100]. The hyaluronate lyase of SF1 has a high homology with the HylB of *S. agalactiae*, which is known to be able to break down hyaluronan and thus is directly involved in host invasion by facilitating the bacterium to overcome the host's physical defense [101]. The streptodornase of SF1 shares 58% sequence identity with the mitogen factor 3 of *S. pyogenes*, which is an extracellular DNase in most clinically isolated *S. pyogenes* strains [102]. In *Pseudomonas aeruginosa*, this protein is reported to be involved in urinary tract infection by removing biofilm [103]. The neuraminidase of SF1 has a 50% sequence identity with the neuraminidase A of *Streptococcus pneumonia*. This enzyme can cleave terminal sialic acids, such as mucin, glycolipids, and, glycoproteins, from host cell surface glycans and lead to enhanced adhesion of the bacteria to host tissues [101], [104].

### Expression profile of the extracellular proteins of SF1 induced by host serum

**Figure 5:**
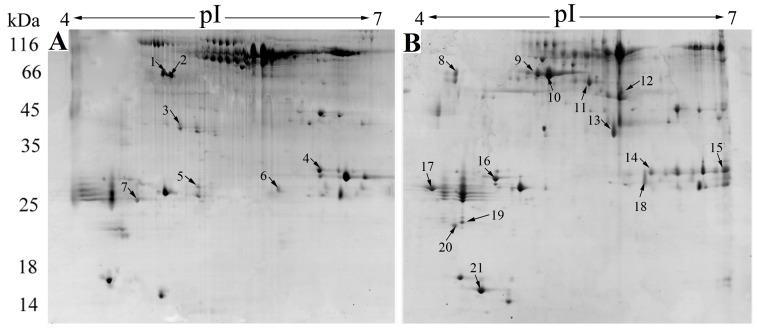
Representative 2-DE maps of the extracellular proteins of *Streptococcus iniae* SF1 treated with or without fish serum. Extracellular proteins were prepared from untreated SF1 (A) and serum-treated SF1 (B) and subjected to 2-DE analysis. Numbers indicate protein spots with differential expression.

With the availability of the complete genome sequence information, which greatly facilitates global transcriptomic and proteomic analysis, we wanted to identify extracellular factors in SF1 that were upregulated during encountering with host defense factors. For this purpose, we examined the expression profile of the extracellular proteins of SF1 treated with or without fish serum. The results, as revealed by the 2-DE maps (Fig. 5), showed that 21 protein spots exhibited apparently differential expressions after serum treatment, with 14 proteins being significantly upregulated (ratio of serum^+^/serum^−^ ≥2, *P*≤0.05) and 7 proteins being significantly downregulated (ratio of serum^−^/serum^+^ ≥2, *P*≤0.05). Of these proteins, five were identified by MALDI-TOF/TOF as enolase (Eno), neuraminidase (Neu), Fe^3+^-siderophore transport protein (Stp), hemolysin (Hem), and hypothetical secreted protein (Hyp1) respectively (Table 5). As said above, enolase is a secreted protein that is known to interact with plasminogen and to assist bacterial adhesion to host cells [99], [100], while neuraminidase is a glycoside hydrolase that plays an important role in pathogenicity by cleaving sialic acids on host cells [101], [104]. Hemolysins are classical virulence factors that cause destruction of red blood cells, whereby contributing to invasion and survival of pathogenic bacteria in the host. Fe^3+^-siderophore transport protein contains a leucine-rich repeat (LRR) domain and two NEAT domain locating at the termini of the protein. The NEAT domain is present in a group of iron-regulated surface proteins found exclusively in bacteria and may contribute to the pathogenicity of most Gram-positive bacterial species [105]. It is likely that upregulated expression of these proteins in SF1 upon contact with host serum is a virulence mechanism of *S. iniae* to achieve optimal infection.

**Table 5:** Five serum-induced extracellular proteins of *Streptococcus iniae* SF1 identified by MALDI-TOF/TOF.

Spotno.^a^	Gene ID	Protein name	Protein score^b^	Isoelectric point	Protein mass	Coverage rate^c^
17	K710_1291	Enolase	51.1	4.36	47231.56	44.78%
14	K710_1065	Predicted secreted protein	50.2	5.82	33025.13	43.15%
15	K710_1941	Neuraminidase	73.4	6.86	109104.22	38.88%
10	K710_1797	Fe^3+^-siderophore transport	61.3	6.77	140620.05	32.43%
21	K710_0586	Hemolysin	69.4	6.64	31560.22	57.35%

a Spot no. represents the numbers on the 2-DE gels (Figure 5).

b MOWSE score is -10 log (p), where p is the probability that the observed match is a random event. Based on the NCBInr database, the MASCOT was used to search program as MS/MS data. Scores greater than 46 are significant (*P* < 0.05).

c Number of amino acid spanned by the assigned peptides divided by the protein sequence length.

### Potentials of serum-induced proteins as candidate vaccines

**Figure 6:**
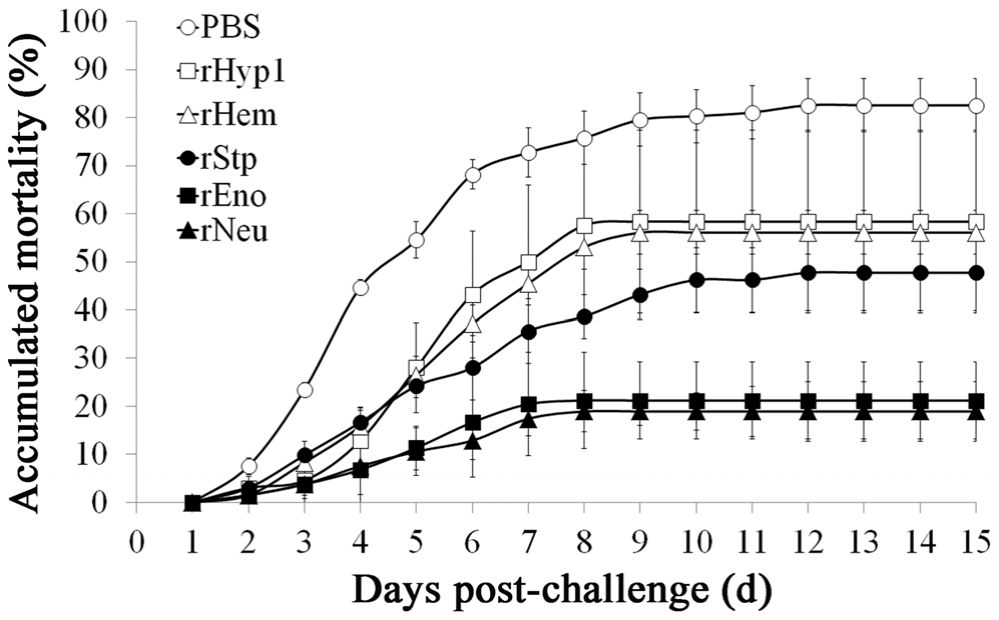
Mortality curves of vaccinated fish. Turbot were vaccinated with rEno, rNeu, rHyp1, rStp, rHem, or PBS (control) and monitored for mortality after challenge with *Streptococcus iniae* SF1. The values in the figure represent the mean mortality values of four vaccination trials.

**Figure 7:**
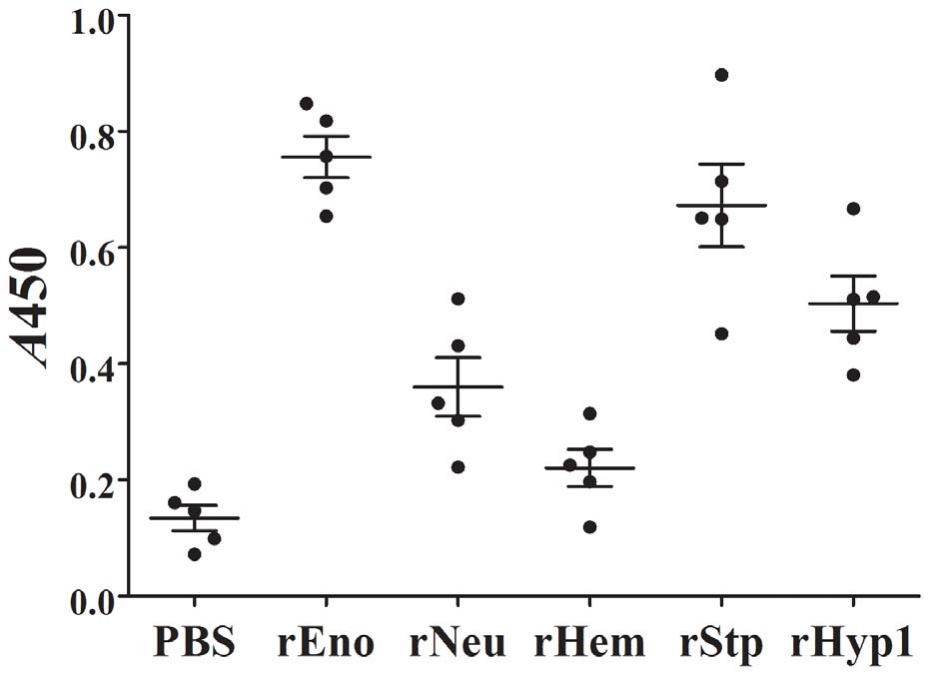
Serum antibody production in vaccinated fish. Sera were taken from fish vaccinated with rEno, rNeu, rHyp1, rStp, rHem, or PBS (control). Specific serum antibodies against each of the proteins were determined by ELISA. Values are shown as means ± SE (*N*  =  5).

Since the above-identified proteins are induced by host serum and secreted to the extracellular milieu, qualities that are likely to enable these proteins to elicit effective host immune response during infection, we examined the potentials of these proteins as subunit vaccines. To this end, recombinant proteins of Eno, Neu, Hem, Stp, and Hyp1 were expressed in and purified from *E. coli* (Supplementary Figure S1). Turbot were immunized with the purified proteins and challenged with a lethal dose of SF1 at four-week p.v.. The vaccination experiment was conducted twice at different times, one without replicate and the other with three replicates. The results showed that the mean accumulated mortalities of the fish vaccinated with rEno, rHyp1, rNeu, rStp, and rHem were 21.2%, 58.3%, 18.9%, 47.7%, and 56.1% respectively, while the mean accumulated mortality of the control fish was 82.6% (Fig. 6, Table S1). Based on these results, the protection rates, in terms of PRS, of rEno, rHyp1, rNeu, rStp, and rHem were 74.3%, 29.4%, 77.1%, 52.2%, and 33.1% respectively. Hence, rEno, rNeu, rHyp1, and rStp induced moderate to high levels of immunoprotection, and therefore may be used as candidate vaccines. These observations are in line with previous reports, which showed that the enolase of *S. pneumoniae* is immunogenic and elicits protective antibody response, and that the enolases of *Streptococcus sobrinus* and *S. suis* confer protections against the respective bacteria [106]–[108]. Neuraminidase is widely used as a vaccine against influenza virus [109], [110] and has been demonstrated to afford protection against *S. pneumoniae*-induced otitis media [109]–[111]. In our study, examination of moribund fish indicated that SF1 was the only type of bacterium isolated from the liver, spleen, and blood, suggesting that mortality was caused by SF1 challenge. ELISA analysis showed that specific serum antibodies were detected in fish vaccinated with rEno, rNeu, rHyp1, and rStp but not in fish vaccinated with rHem (Fig. 7). The inability to activate B cell-mediated immune response may account at least in part for the failure of rHem to induce effective protection.

## Supporting Information

Figure S1 **SDS-PAGE analysis of purified recombinant proteins.** Purified rHem, rHyp1, rEno, rNeu, and rStp (lanes 1 to 5 respectively) were analyzed by SDS-PAGE and viewed after staining with Coomassie brilliant blue R-250. M, protein markers.
(TIF)

Table S1 **Summary of the vaccination results.**
(DOC)
