# Supplementary figures and images for: Streptococcus iniae SF1: Complete Genome Sequence, Proteomic Profile, and Immunoprotective Antigens
Source: PLoS One. 2014 Mar 12;9(3):e91324. doi: 10.1371/journal.pone.0091324 (PMC3951389; doi:10.1371/journal.pone.0091324)

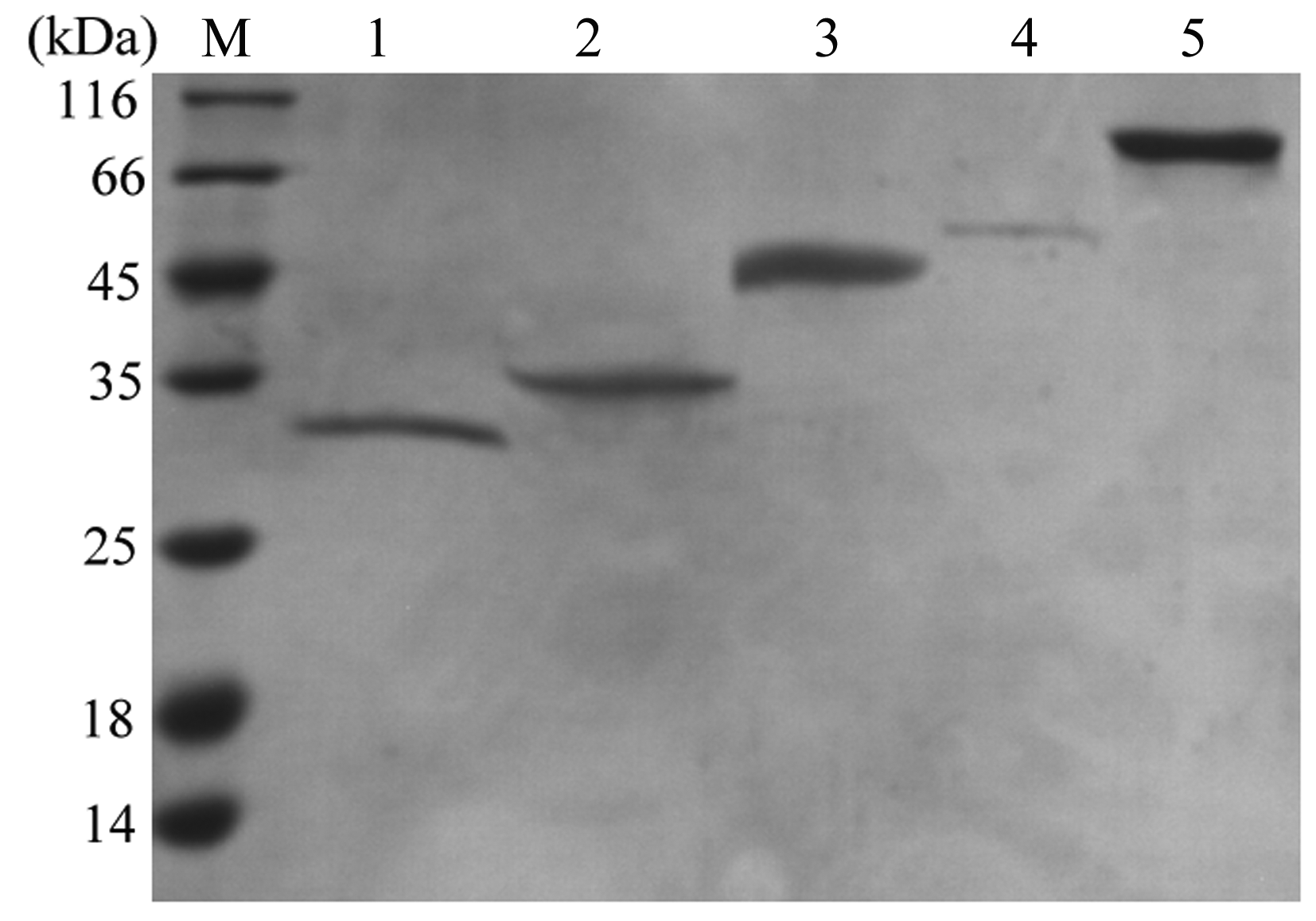

Supplement: Figure S1 — SDS-PAGE analysis of purified recombinant proteins. Purified rHem, rHyp1, rEno, rNeu, and rStp (lanes 1 to 5 respectively) were analyzed by SDS-PAGE and viewed after staining with Coomassie brilliant blue R-250. M, protein markers. (TIF) [file pone.0091324.s001.tif]
