# Supplementary material for: Streptococcus iniae SF1: Complete Genome Sequence, Proteomic Profile, and Immunoprotective Antigens
Source: PLoS One. 2014 Mar 12;9(3):e91324. doi: 10.1371/journal.pone.0091324 (PMC3951389; doi:10.1371/journal.pone.0091324)
Supplement: Table S1 — Summary of the vaccination results. (DOC) [file pone.0091324.s002.doc]

**Table S1.** Summary of the vaccination results.

| Vaccine | Cumulative mortality (%) | | | | |  | RPS^b^ (%) |
| --- | --- | --- | --- | --- | --- | --- | --- |
|  | I^a^ | II^a^ | III^a^ | IV^a^ | Mean |  |  |
| PBS | 78.8 | 87.9 | 87.9 | 75.8 | 82.6 |  | - |
| rEno | 18.2 | 24.2 | 27.3 | 15.2 | 21.2 |  | 74.3 |
| rHyp1 | 30.3 | 72.7 | 66.7 | 63.6 | 58.3 |  | 29.4 |
| rNeu | 15.2 | 21.2 | 15.2 | 24.2 | 18.9 |  | 77.1 |
| rStp | 48.5 | 54.5 | 36.4 | 51.5 | 47.7 |  | 52.2 |
| rHem | 63.6 | 60.6 | 45.5 | 54.5 | 56.1 |  | 32.1 |

^a^I, II, III, and IV represent the results of four vaccination trials; ^b^RPS, relative percent of survival.
